# Effect of Maturity on Phenolics (Phenolic Acids and Flavonoids) Profile of Strawberry Cultivars and Mulberry Species from Pakistan

**DOI:** 10.3390/ijms13044591

**Published:** 2012-04-11

**Authors:** Tahir Mahmood, Farooq Anwar, Mateen Abbas, Nazamid Saari

**Affiliations:** 1 Department of Chemistry & Biochemistry, University of Agriculture, Faisalabad-38040, Pakistan; E-Mail: ranatahiruaf@yahoo.com; 2 Department of Chemistry, Govt. Post Graduate College Samanabad, Faisalabad-38040, Pakistan; 3 Department of Chemistry, University of Sargodha, Sargodha-40100, Pakistan; 4 Quality Operation Laboratory, University of Veterinary and Animal Sciences-54000, Lahore, Pakistan; E-Mail: hafizmateen2002@yahoo.com; 5 Faculty of Food Science and Technology, Universiti Putra Malaysia, UPM-43400 Serdang, Selangor, Malaysia

**Keywords:** small fruits, TPC, TFC, quercetin, kaempferol, *p*-coumaric acid, fruit ripening, *p*-hydroxybenzoic acid, HPLC

## Abstract

In this study, we investigated how the extent of ripeness affects the yield of extract, total phenolics, total flavonoids, individual flavonols and phenolic acids in strawberry and mulberry cultivars from Pakistan. In strawberry, the yield of extract (%), total phenolics (TPC) and total flavonoids (TFC) ranged from 8.5–53.3%, 491–1884 mg gallic acid equivalents (GAE)/100 g DW and 83–327 mg catechin equivalents (CE)/100 g DW, respectively. For the different species of mulberry the yield of extract (%), total phenolics and total flavonoids of 6.9–54.0%, 201–2287 mg GAE/100 g DW and 110–1021 mg CE/100 g DW, respectively, varied significantly as fruit maturity progressed. The amounts of individual flavonols and phenolic acid in selected berry fruits were analyzed by RP-HPLC. Among the flavonols, the content of myricetin was found to be high in *Morus alba* (88 mg/100 g DW), the amount of quercetin as high in *Morus laevigata* (145 mg/100 g DW) while kaempferol was highest in the Korona strawberry (98 mg/100 g DW) at fully ripened stage. Of the six phenolic acids detected, *p*-hydroxybenzoic and *p*-coumaric acid were the major compounds in the strawberry. *M. laevigata* and *M. nigra* contained *p*-coumaric acid and vanillic acid while *M. macroura* and *M. alba* contained *p*-hydroxy-benzoic acid and chlorogenic acid as the major phenolic acids. Overall, a trend to an increase in the percentage of extraction yield, TPC, TFC, flavonols and phenolic acids was observed as maturity progressed from un-ripened to fully-ripened stages.

## 1. Introduction

Soft fruits such as strawberries and mulberries are gaining greater recognition among other fruit crops due to their high economic and nutritional value. Recently, these fruits have gained much attention as an ingredient of functional foods due to their potential source of valuable bioactives such as flavonoids, phenolic acids and free radical scavengers with potential health benefits [1,2]. The quality of soft fruits, in terms of taste, functional food value and consumer’s acceptance, is primarily based on their biochemical composition [3,4].

Flavonoids are broadly dispersed in the plant kingdom accounting for over half of the 8000 naturally occurring phenolic compounds [5]. Among the phytochemicals in fruit, phenolic acids and flavonols are regarded as major functional food components and are thought to contribute to the health effects of fruit-derived products due to the prevention of various diseases associated with oxidative stress, such as cancers, cardiovascular diseases and inflammation [6,7] Phenolic acids constitute about one-third of the dietary phenols and are present in plants in free and bound forms [8].

Maturation of fruit or other plant tissues involves a series of complex reactions, which leads to changes in the phytochemistry of the plants. Two distinct phenomena of change in phenolic contents have been observed during maturation: Steady decrease [9,10] or rise at the end of maturation [11–14]. The content of phenolics in fruit is affected by the degree of maturity at harvest, genetic differences (cultivar), pre-harvest environmental conditions, and post-harvest storage conditions and processing [15], however, their concentration varies from plant to plant or even in different organs of the same plant at different ripening stages [16,17].

The commercial strawberry fruit (*Fragaria* × *ananassa* Duch.) belongs to the *Rosales* order of the *Rosaceae* family [18]. It is one of the most widely consumed fruits worldwide, either as a fresh fruit, as processed products or even as dietary supplements. Worldwide, the production of strawberries has increased steadily during the last 40 years with most (>95%) of it being located in the northern hemisphere. The USA is the leading producer, followed by Spain, Turkey and the Russian Federation. China is nowadays a direct competitor for most of the major strawberry producing regions with an estimated production of 1.3 million metric tons (MMT) for the period of 2010 [19]. Strawberry growth and development is characterized by changes in color, size, sweetness, acidity, and aroma [20,21]. Four or five different maturity stages for strawberry fruit are described in the literature according to the development of the non-ovarian receptacle tissue [22,23].

Marked compositional variability in the content of phenolics in berries is not only affected by varietal or cultivar, genetic differences, season, and climate, but also by the degree of maturity at harvest [24–26].

Mulberry belongs to the genus *Morus* of the family *Moraceae. Morus* have 24 species with one subspecies and comprise at least 100 known varieties. Black (*M. nigra*), red (*M. rubra*), and white mulberries (*M. alba*) are extensively grown in Pakistan, northern India, and Iran. These are known by the Persian-derived names *toot* (mulberry) or *shahtoot* (King’s or “Superior” mulberry). *Shahtoot* (*M. laevigata*), particularly the white variety, is a popular hybrid species in Pakistan. Mulberries are grown at considerably high altitudes in the Himalaya-Hindu Kush region and are widely cultivated in northern regions of Pakistan [27,28]. In Pakistan, *shahtoot* is valued for its delicious fruit, which is eaten fresh as well as in dried forms, and consumed in marmalades, juices, and liquors, and used for natural dyes and in the cosmetic industry [29]. The deep colored mulberry fruits are rich in phenolic compounds, including flavonoids, carotenoids and anthocyanins [30–32].

Previous studies mostly conducted on ripened strawberry fruit reported significant amounts of phenolic acids and flavonols. The major phenolic acids in strawberry are neochlorogenic acid and *p*-coumaryl quinic acid [33] as hydroxycinnamic acids derivatives [34,35]. However, small amounts of chlorogenic acid [33] and ferulic acid [36] have also been reported. Hydroxybenzoic acids (*p*-hydroxybenzoic acid) were only found in small amounts in strawberry [36]. According to Franke *et al*. [37] and Olsson *et al*. [38], kaempferol was detected to be the major flavonol while Sultana and Anwar [39] reported myricetin to be the main flavonol compound in selected cultivars of strawberry.

Studies of ripening are of special interest because they allow the identification of the optimum point of maturity for harvesting and enable delivery of fruit to consumers in its best condition in terms of nutritional and functional properties. Information regarding the changes in particular phenolic constituents during fruit maturation is limited. In this study, we looked at how the accumulation of phenolic acids and flavonol in the strawberry and mulberry fruits is affected by maturation. The results will be informative and novel with regard to the quantification of specific flavonols and fruit materials considering their native region and the effect of maturity. This study will be valuable for researchers in providing base line data for future detailed characterization of other bioactives in these fruits, and thus a step forward towards their potential commercialization for nutraceutical and anti-oxidant applications through value addition.

## 2. Results and Discussion

### 2.1. Effect of Maturity on the Yield of Extract (%), Total Phenolics and Total Flavonoid Content in Strawberry and Mulberry Fruits

The results showed that the yield of extract (%), total phenolics (TPC) and total flavonoid content (TFC) in the strawberry and mulberry fruit cultivars at different maturity stages varied considerably (Table 1). As the fruit maturity progressed, the yield of extract (%), TPC and TFC of strawberry fruit increased from 8.5 to 53.3%, 491 to 1884 mg gallic acid equivalents (GAE)/100 g DW and 83–327 mg CE/100 g DW, respectively. Similar to our present finding, an increasing trend in total phenolics (216–290 mg GAE/100 g FW) as fruit maturity progressed in two strawberry cultivars has been reported by Pineli *et al*. [13]. Bohm *et al*. [40] found a TPC between 1800–2200 mg GAE/100 g while Piljac-Zegarac and Samec [41] reported values as high as 335 mg GAE/100 g FW in ripe strawberries. In another study conducted by Lin and Tang [32], the TFC (14.6 mg QE/100 g FW) of ripened strawberries was found to be in close agreement with that determined in fully ripened samples of the present work.

Significant variation was also observed in the yield of extract (%), TPC and TFC of mulberry fruit. The highest yield of extract (%) was obtained for *M. laevigata* (12–54%) while the lowest was found for *M. nigra* (11–28%). The concentration of total phenolics (TP) was highest in *M. nigra* (395–2287 mg GAE/100 g DW) while it was lowest in *M. laevigata* (201–1803 mg GAE/100 g DW). Regarding TFC, *M. nigra* contained the highest (245–1021 mg CE/100 g DW) and *M. macroura* the lowest levels (145–249 mg CE/100 g DW).

TPC (223–257 mg GAE/100 g DW) and TFC (0.06–6.54 mg CE/100 g DW) as studied by Bae and Suh [42] in five Korean mulberry cultivars (Pachungsipyung, Whazosipmunja, Suwonnosang, Jasan, and Mocksang) were somewhat lower than our present results. Lin and Tang [32] found that *Morus alba* had 1515 mg GAE/100 g DW of TP. Similarly, in another study by Ercisli and Orhan [28], the amount of TP in different species of mulberry fruit varied from 181 (*M. alba*)–1422 (*M. nigra*) mg GAE/100 g FW and the total flavonoids (TF) from 29 (*M. alba*) to 276 (*M. nigra*) mg QE/100 g FW. As investigated previously by Imran *et al*. [43], the contents of TP in *M. laevigata* varied considerably (1100–1300 mg/100 g FW). TPC of the *Morus alba* fruit from Turkey ranged from 18.16 to 19.24 μg GAE/mg [44]. With few exceptions, these results are all within the range of our present data. The difference of phenolics (TPC and TFC) among different mulberry fruits might be linked to their varied genetic makeup as well as the extent of fruit maturity and ecological conditions of the harvest [26]. It has previously been reported that plant genotype [45], cultivation site and extraction technique [46] affect the total phenolic contents in berry group fruits.

Overall, a trend towards increase was observed in the yield of extract (%), TPC and TFC as strawberry and mulberry fruits progressed from un-ripened to fully-ripened stages. Likewise, Aminah and Anna [47] described the effect of different ripening stages on bitter gourd and observed an increase in TP as maturity progressed. In agreement to our findings, several authors reported an increase in the concentration of TP in different fruits such as Khirni [12], sweet cherry [11], *Morinda citrifolia* [14] and strawberry [13] as maturity progressed. However, an inverse trend for TPC was reported by some other authors in mushrooms [48] and strawberry fruits [9,10].

### 2.2. Effect of Maturity on Quantification of Flavonols and Phenolic Acid

**Figure 1:**
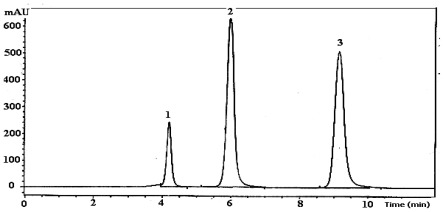
Typical HPLC chromatogram of standard flavonol mixture; peak identification: 1. Myricetin (RT 4.176); 2. Quercetin (RT 5.969); 3. Kaempferol (RT 9.130).

**Figure 2:**
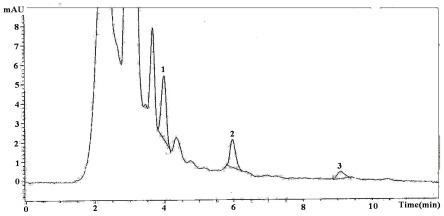
A typical HPLC chromatogram of strawberry (Korona) samples at fully ripened stage showing separation of flavonols; Peak Identification: 1. Myricetin (RT 4.176) 2. Quercetin (RT 5.969); 3. Kaempferol (RT 9.130).

The data for the quantitative analysis of individual flavonols and phenolic acids in strawberry cultivar fruits at different maturity stages are presented in Table 2. Kaempferol was the dominant flavonol in strawberry followed by myricetin and quercetin (Figures 1,2). Kaempferol levels in the strawberry cultivar fruit during three maturity stages ranged from 19.9 to 98.1 mg/100 g DW. The kaempferol content of strawberry fruit in the present investigation was found to be higher than that previously reported, namely 0.6 to 1.3 mg/100 g [37], 10.8–43.7 mg/100 g [38] in fully-ripened strawberry fruits. In the present analysis of strawberry, the amounts of myricetin and quercetin varied from 12.8–28.5 and 1.4–11.2 mg/100 g DW, respectively.

In strawberry (Var. Korona and Tufts) the contents of flavonols (kaempferol, myricetin and quercetin) were mainly increased as the fruit maturity progressed from un-ripened to fully-ripened stages. Lugasi and Hovari [49] found that quercetin was present in strawberry at 5.3 mg/100 g whereas myricetin was present at 99.4 mg/100 g and kaempferol was not detected in strawberry samples. Cordenunsi *et al*. [3] reported the contents of quercetin and kaempferol in three strawberry cultivars to be in the range of 3.9–6.8 mg/100 g and 1.3–2.1 mg/100 g FW, respectively. Kevers *et al*., [50] described that strawberry contained kaempferol, quercetin and myricetin at the levels of 99, 123 and 979 μg/100 g FW, respectively. Fruits are an important source of dietary polyphenols in human nutrition and contribute significantly to the daily intake of polyphenols (32% of the daily intake of flavonols in Finland) [51]. Studies revealed that the total polyphenols (12–50 mg/g DW) in fruit is much higher than in vegetables (0.4–6.6 mg/g DW) and cereals (0.2–1.3 mg/g DW) [52].

**Figure 3:**
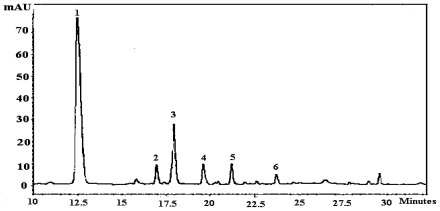
Typical HPLC chromatogram of standard phenolic acid mixture; peak identification: 1. Gallic acid; 2. Chlorogenic acid; 3. *p*-hydroxy-benzoic acid; 4. Vanillic acid; 5. *p*-coumaric acid; 6. Ferulic acid.

**Figure 4:**
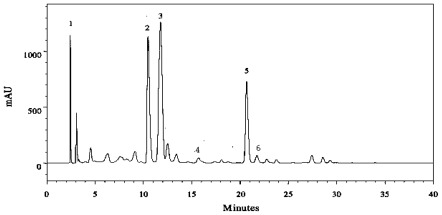
Typical HPLC chromatogram of strawberry (Korona) at semi-ripened stage showing separation of phenolic acids; peak identification: **1**. Gallic acid; **2**. Chlorogenic acid; **3**. *p*-hydroxy-benzoic acid; **4**. Vanillic acid; **5**. *p*-coumaric acid; **6**. Ferulic acid.

The amount of individual phenolic acids in the tested fruits varied significantly (*p* < 0.05) in relation to different maturity stages. The major phenolic acids found in strawberry were *p*-hydroxybenzoic acid and *p*-coumaric acid (Figures 3, 4). The concentration of *p*-hydroxybenzoic acid in strawberry cultivars ranged from 21.4–65.4 mg/100 g DW, followed by *p*-coumaric (17.3–47.5 mg/100 g DW), gallic (6.8–24.6 mg/100 g DW), ferulic (7.6–24.1 mg/100 g DW), chlorogenic (11.5–18.2 mg/100 g DW) and vanillic (2.8–16.1 mg/100 g DW) acids from un-ripened to fully-ripened stage.

Previous studies reported methanol soluble cinnamic acid and *p*-hydroxycinnamic acid in strawberries to be the major components followed by caffeic acid and ferulic acid [2,51]. In another study, *p*-coumaric acid was found to be the predominant hydroxycinnamic acid as sugar esters in strawberries and raspberries and as free form in cloudberries [53]. As reported above [54], *p*-hydroxybenzoic and *p*-hydroxycinnamic were the most abundant phenolic acids in strawberry fruit, and occurred in almost equal quantities (ranging from 64.9–110.5 mg/100 g and 64.2–110.4 mg/100 g, respectively), which is comparable with our present results.

The amount of *p*-coumaric acid notably increased during maturity in strawberry cultivars [55]. The concentrations of chlorogenic and *p*-coumaric acids also increased during ripening of strawberry [38]. Ndri *et al*. [56] studied the phenolics in Ivorian Gnagnan (*Solanum indicum* L.) berries at different maturity stages and found that as the maturity progressed, the amount of phenolic acids increased. These trends are similar to those displayed in our present study.

Hybrid strawberry cultivated in Turkey [55] contained 4–58 mg/kg FW of *p-*coumaric acid, while Ecuador strawberry [57] contained 18 mg/kg FW. In another study of six Finnish strawberry types [46], the content of *p-*coumaric acid was 9–41 mg/kg FW showing comparable values with our present study. Hernanz *et al*. [58] assessed statistically significant differences (*p* < 0.001) of phenolic acids among five strawberry cultivars grown in two different soilless systems. Ellagic and *p*-coumaric acids were the major phenolic acids found in the Finnish strawberry as reported by Hakkinen *et al*. [51]. Similar results were reported by Maatta-Riihinen *et al*. [53] and Cordenunsi *et al*. [3] in a commercial strawberry harvested from Brazil. In the case of *p*-coumaric acid, its level varied from 1.43 μg/g (*cv.* Diamante-CS) to 25.47 μg/g (*cv*. Ventana-CS) [58].

When comparing flavonoids, the cultivars analyzed in the present study were more promising in relation to beneficial effects on health, due to their higher content of flavonols. A wide variation of flavonoids in strawberry cultivars has been reported in the literature. These variations may be correlated to the varying genetic makeup of the varieties tested as well as to the post harvest conditions involved. In another related study, the effect of storage conditions on the flavonoid content was investigated, and the amount of quercetin was found to be increased while kaempferol and myricetin were decreased during storage at −20 °C [59]. Variation in flavonol content in fruits is strongly influenced by extrinsic factors such as fruit type and growth, season, climate, degree of ripeness, food preparation, and processing [60–63].

The data in Table 3 depicts the composition of flavonols and phenolic acids of mulberry fruits at different maturity stages. *Morus laevigata* had the highest amount of total flavonols (quercetin myricetin, kaempferol) followed by *Morus nigra*, *M. alba* and *M. macroura.* Kaempferol and quercetin amounts were highest in *M. laevigata* while myricetin was predominant in *M. alba*. The concentration of kaempferol increased with ripening, ranging from 9.8 mg/100 g (un-ripened) to 56.1 mg/100 g (fully-ripened) and quercetin ranged from 7.0 mg/100 g (un-ripened) to 145.7 mg/100 g (fully-ripened) for *M. laevigata.* The myricetin content increased from un-ripened to semi-ripened stages (11.5–22.3 mg/100 g), and then slightly decreased at the fully-ripened stage (20.0 mg/100 g). In *M. macroura* and *M. alba*, the concentration of kaempferol was decreased from the un-ripened to fully-ripened stage while the reverse trend was observed for myricetin. Meanwhile, *M. alba* showed a decreasing trend (1.3–0.7 mg/100 g) as the fruit progressed from un-ripened to fully-ripened stage. The level of flavonols (kaempferol, quercetin, myricetin) in *M. nigra* increased from un-ripened to semi-ripened stage (8.56–56.62, 8.10–43.46, 52.57–63.30 mg/100 g) and then decreased at fully-ripened stage (31.67, 11.75, 56.10 mg/100 g).

Compositional changes of flavonols during ripening due to several biotic and abiotic factors significantly affected their accumulation in berries and grapes [64]. Consequently, the time when the fruit is picked has a strong impact on the flavonol content. Bilyk and Sapers [65] reported a positive correlation between flavonol contents and blackberry maturity from red to black (quercetin content 9.01–15.8 mg/100 g FW and kaempferol content 0.7–1.74 mg/100 g FW). In another study conducted by Vuorinen *et al.* [63], the level of flavonol glycosides in black currants was increased significantly during berry ripening. With increasing degree of ripening, the content of quercetin and kaempferol was found to be enhanced for both the investigated years in strawberry cultivar Honeoye, whereas a smaller difference was seen in the cultivar Senga Sengana [38]. The above reported studies by different authors support our present findings which reveal that as the maturity progresses the contents of flavonol also increases.

The major phenolic acids found in mulberry species were: *p*-coumaric acid, chlorogenic acid and *p*-hydroxybenzoic acid (Table 3). *Morus laevigata* and *M. nigra* contained higher amounts of *p*-coumaric acid and vanillic acid while *M. macroura* and *M. alba* showed *p*-hydroxy-benzoic acid and chlorogenic acid as the major phenolic acids. The overall trends of phenolic acids in mulberry species were similar to those recorded for strawberry (Table 2). The concentration (mg/100 g DW) of vanillic acid increased as maturity progressed from un-ripened to fully-ripened stages in the tested mulberry species: *M. laevigata* (8.5–21.1) *M. macroura* (3.2–16.1) *M. alba* (1.7–5.7) and *M. nigra* (6.1–18.3), respectively. Among different mulberry species, *M. laevigata* was found to be higher in *p*-coumaric, ferulic, *p*-hydroxy-benzoic, chlorogenic and gallic acids with a contribution of 15.9–27.3, 12.4–17.2, 1.1–7.3, 3.4–12.9 and 5.2–14.2 mg/100 g DW, respectively at un-ripened to fully-ripened stages.

*M. macroura* was found to be rich in *p*-coumaric (5.1–13.2 mg/100 g DW), ferulic (6.3–13.4 mg/100 g DW), *p*-hydroxy-benzoic (5.1–24.1 mg/100 g DW), chlorogenic (4.2–23.2 mg/100 g DW) and gallic acid (4.2–9.8 mg/100 g DW) from un-ripened to fully ripened stage. *M. alba* also contained slightly lower amounts of these phenolic acids except for ferulic acid which was not found in this cultivar. *Para*-coumaric, ferulic, chlorogenic and gallic acids in *M. nigra* were in the range of 4.2–21.2, 2.4–7.5, 2.5–6.8 and 2.5–8.3 mg/100 g DW, respectively, while *p*-hydroxy-benzoic acid (5.3 mg/100 g DW) was detected only at fully-ripened stages.

Memon *et al*. [66] described the composition of phenolic acids in mulberry (*Morus laevigata* W., *M. nigra* L., *M. alba* L.) fruits grown in Pakistan: Chlorogenic (20.5 mg/100 g) and *p*-hydroxybenzoic acids (15.3 mg/100 g) were the predominant compounds in *M. alba* whereas *p*-coumaric acid (8.7 mg/100 g) was found to be higher in *M. nigra*. However, different phenolic acids were evenly distributed in *M. laevigata*. These data on *Morus* species are in agreement with those we determined in the present analysis.

Phenolic compounds are important bioactives and their content in fruits represents an important fruit quality parameter [67]. Some earlier studies [33,51,55] showed that consumption of the strawberry and mulberry fruits may have a positive impact on the human health, which might be linked to the amounts of polyphenolics in these fruits. The increasing importance of functional ingredients in food pushes plant sciences to increase health-promoting phytochemicals in fruit crops. Higher intakes of flavonoids and other antioxidant compounds from food are associated with a reduced risk of cancer, heart disease, and stroke. Some experimental studies indicate that several plant flavonols, such as quercetin, myricetin, and rutin, are more powerful antioxidants than traditional vitamins and have antitumor properties. The challenge is how to increase the levels of these beneficial phytochemicals in different foods for optimal nutrition. Currently the use of modern biotechnological techniques, such as genetic engineering, to produce transgenic plants with enhanced amounts of valuable bioactives [68] as well as exogenous applications of organic osmolytes, such as glycerinbetaine and proline, to increase the levels of antioxidant and phenolics in different food crops [69,70] are fascinating.

## 3. Experimental Section

### 3.1. Collection of Samples

In this study, fruit samples of strawberry (*Fragaria* × *ananassa* Duch) cultivars (Korona and Tufts) and mulberry (*M. alba, M. nigra*, *M. macroura, M. laevigata*) at un-ripened, semi-ripened and fully-ripened stages were collected from the Lahore and Faisalabad region during April-July, 2009. The selection of the fruits at different maturity stages was based upon their color and texture (Table 4). The fruits of strawberry and mulberry were hot air dried to constant mass. The dried samples were ground (80 mesh size) and then preserved in polyethylene bags. Three different samples for each of the fruit cultivar at each maturity stages were assayed.

### 3.2. Reagents

In the research work, *p*-coumaric, vanillic, chlorogenic, *p*-hydroxybenzoic, ferulic and gallic (phenolic acids standards), kaempferol, quercetin, and myricetin (flavonol standards), and ter-butylhydroquinone (TBHQ) were acquired from Sigma-Aldrich (St Louis, MO, USA). HPLC grade methanol, acetonitrile and all other chemicals used in this study were purchased from Merck (Darmstadt, Germany). Stock samples of flavonol and phenolic acids were prepared in methanol at concentrations of 200 mg/L. Working samples were diluted with the corresponding mobile phase to 10 mg/L. Samples were passed through a 0.45 μm nylon filter membrane (MSI) before injection. Both stock and working samples were stored in a refrigerator at 4 °C in darkness. The calibration curves were constructed using peak area *vs*. concentration.

### 3.3. Dry Matter Determination

In view of varying degrees of fruit moisture among the species analyzed, all calculations were made on a dry matter basis. Dry matter determination was made according to AOAC procedure (method 925.10). Briefly, 5 g of the sample was dried in an electric oven at 105 °C until a constant weight was recorded.

### 3.4. Sample Extraction for Antioxidant Assay

The ground material (10 g) of strawberry and mulberry fruit at each maturity stage was extracted separately with 100 mL of 80% aqueous methanol (80:20) for 6 h at room temperature in an orbital shaker (Gallenkamp, UK). The extracts were separated from the residues by filtering through Whatmann No. 1 filter paper. The residues were re-extracted twice with the same fresh solvent. The recovered extracts were combined and freed of solvent under reduced pressure at 45 °C using a rotary evaporator (EYELA, SB-651, Rikakikai Company Limited, Tokyo, Japan). The crude extracts were quantitatively transferred into a sample vial and stored in a refrigerator until used for further experiments.

### 3.5. Determination of Total Phenolics Content (TPC)

The amount of total phenolics was determined by using the previously mentioned method of Chaovanalikit and Wrolstad [71], with slight changes. Briefly, the crude extract (1 mg) was mixed with tenfold diluted 2 N Folin-Ciocalteu reagent (1.0 mL) and 0.5 mL de-ionized water. The mixture was kept at room temperature for 10 min, and then 0.8 mL of Na_2_CO_3_ (7.5% *w/v*) was added. The mixture was heated in a water bath at 40 °C for 20 min and then cooled in an ice bath; absorbance was measured at 760 nm using a spectrophotometer. Amounts of TP were calculated using a gallic acid calibration curve within the concentration range of 10–100 ppm (*R*^2^ = 0.9986). The results were expressed as gallic acid equivalents (GAE mg/100 g DW). All samples were analyzed thrice and results averaged.

### 3.6. Determination of Total Flavonoids Content (TFC)

The total flavonoids were measured colorimetrically following a previously reported method [72]. In summary, the crud extract (5 mg) of each selected fruit was diluted with 5 mL distilled water in a 10 mL test tube. Initially, 0.3 mL of 5% NaNO_2_ was added to each test tube; after 5 min, 0.6 mL of 10% AlCl_3_ was added; after 5 min, 2 mL of 1.0 M NaOH was added. Absorbance of the reaction mixture was measured at 510 nm using a spectrophotometer. TFC were determined as catechin equivalents (CE mg/100 g DW). Three readings were taken for each sample and results averaged.

### 3.7. Extraction and Hydrolysis for Quantification

Extraction/hydrolysis of flavonols and phenolic acids was carried out using the method described by Sultana and Anwar [39]. In summary, 25 mL of acidified methanol containing 1% (*v/v*) HCl and 0.5 mg/mL BHT as an antioxidant were added to the ground fruit material (5 g) in a refluxing flask. Then 5 mL of HCl (1.2 M) was added and the mixture was stirred at 90 °C under reflux for 2 h to obtain aglycons of flavonol glycosides and to convert bound phenolic acids into free forms. The extract was cooled to room temperature and centrifuged at 1500 g for 10 min. The upper layers were taken and sonicated for 5 min to remove any air present in the extract. The final extracts were filtered through a 0.45-μm (Millipore) filter before they were analysed by HPLC. Phenolic acids and flavonols were separated and quantified following HPLC conditions.

### 3.8. Instrumentation

Chromatographic analysis was carried out on an Agilent 1100-series HPLC system equipped with a Quaternary pump (G1311A Quat pump), vacuum degasser (G1379A), auto-sampler/auto-injector (G1313A ALS), column compartment (G1316A Colcom) and DAD detector (G1315B DAD). An Agilent Chem Station was used to process chromatographic data.

### 3.9. Conditions Used for Phenolic Analysis

A Hibar^®^ RP-C18 column (250 mm × 4.6 mm, 5 μ particle size) from Merck Company (Merck KGaA, 64271 Darmstadt, Germany) thermostated at 25 °C was used for separation. The mobile phase (50% tri-fluoroacetic acid (0.3%), 30% acetonitrile and 20% methanol) for flavonols and 40% tri-fluoroacetic acid (0.3%), 40% acetonitrile and 20% methanol for phenolic acids were added at a flow rate of 1.0 mL/min. The mobile phase was filtered through a Nylon membrane filter (47 mm, 0.45 mm) and was degassed by sonication before elution. Isocratic and gradient elution and detection at 360 and 280 nm were selected for separation and detection of flavonols and phenolic acids, respectively. The compound identification was carried out by comparison of their retention times with those of authentic standards. The additional identification was carried out by spiking the extracts with phenolic standards.

### 3.10. Statistical Analysis

Each fruit (strawberry and mulberry) was analysed at each maturity stage and in triplicate. Data were reported as mean ± SD. Analysis of variance (ANOVA) was performed using Minitab 2000 Version 13.2 statistical software (Minitab Inc., State College, PA, USA). A probability value of *p* < 0.05 was considered as a statistically significant difference.

## 4. Conclusions

The quantitative and qualitative differences between TPC and TFC in strawberry and mulberry fruits during ripening were observed, which depend on different fruit cultivars. Mostly, a trend to increasing amounts of these constituents was recorded. Of the fruits analyzed in the present study, *Morus laevigata* and Korona strawberry exhibited commendably higher levels of flavonols and phenolic acids, which support their functional food use. Thus, the results of the present study support the antioxidant and nutraceutical potential of these fruits indigenous to Pakistan. However, further investigations involving more detailed *in-vitro* and *in-vivo* studies are required to ascertain an inclusive phenolic antioxidant system of these fruits and develop their application for specific food or nutraceutical purposes.

## Figures and Tables

**Table 1:** Effect of maturity on % extraction yield, total phenolics and total flavonoids of strawberry and mulberry fruits.

Fruits	Species	Maturity stages	% extraction yield	Total Phenolics A	Total flavonoids B
Strawberry	Korona	Un-ripened	08.9 ± 1.1 ^c^	581 ± 18 ^c^	123 ± 10 ^c^
		Semi-ripened	31.6 ± 3.2 ^b^	938 ± 42 ^b^	174 ± 15 ^b^
		Fully-ripened	53.3 ± 4.8 ^a^	1884 ± 69 ^a^	327 ± 17 ^a^
	Tufts	Un-ripened	08.5 ± 1.6 ^c^	491 ± 22 ^c^	83 ± 06 ^b^
		Semi-ripened	24.4 ± 1.7 ^b^	794 ± 44 ^b^	98 ± 07 ^b^
		Fully-ripened	43.4 ± 2.5 ^a^	1662 ± 88 ^a^	197 ± 07 ^a^
Mulberry	*M. laevigata*	Un-ripened	14.6 ± 1.0 ^c^	201 ± 07 ^c^	304 ± 16 ^c^
		Semi-ripened	33.3 ± 2.7 ^b^	466 ± 17 ^b^	559 ± 34 ^b^
		Fully-ripened	52.3 ± 2.3 ^a^	1803 ± 67 ^a^	615 ± 26 ^a^
	*M. macroura*	Un-ripened	12.3 ± 1.2 ^c^	219 ± 06 ^c^	145 ± 09 ^c^
		Semi-ripened	35.4 ± 3.6 ^b^	508 ± 14 ^b^	282 ± 07 ^a^
		Fully-ripened	54.0 ± 4.2 ^a^	2067 ± 67 ^a^	249 ± 08 ^b^
	*M. nigra*	Un-ripened	11.4 ± 0.8 ^b^	395 ± 17 ^c^	245 ± 06 ^c^
		Semi-ripened	24.0 ± 2.6 ^a^	1722 ± 37 ^b^	706 ± 32 ^b^
		Fully-ripened	28.3 ± 2.1 ^a^	2287 ± 41 ^a^	1021 ± 75 ^a^
	*M. alba*	Un-ripened	06.9 ± 0.7 ^c^	575 ± 12 ^c^	110 ± 07 ^c^
		Semi-ripened	30.8 ± 2.8 ^b^	1071 ± 31 ^b^	392 ± 06 ^b^
		Fully-ripened	40.2 ± 2.0 ^a^	1872 ± 57 ^a^	625 ± 17 ^a^

Values (mean ± SD) are averages of three samples of each fruit, analyzed individually in triplicate (*p* < 0.05); The different small letters in superscript represent the significant differences of ripening stages;

A as per gallic acid equivalent (mg GAE/100 g DW);

B as per catechin equivalent ( mg CE/100 g DW).

**Table 2:** Flavonols and phenolic acids composition (mg/100 g DW) of strawberry fruits at different maturity stages.

	Strawberry (Korona)			Strawberry (Tufts)		
**Flavonols**	Un-ripened	Semi-ripened	Fully-ripened	Un-ripened	Semi-ripened	Fully-ripened
Myricetin	13.2 ± 0.5 ^c^	28.5 ± 1.2 ^a^	20.8 ± 0.5 ^b^	12.8 ± 0.3 ^c^	20.1 ± 0.8 ^b^	23.1 ± 1.4 ^a^
Quercetin	1.4 ± 0.1 ^c^	5.5 ± 0.1 ^b^	5.5 ± 0.1 ^b^	5.9 ± 0.9 ^b^	11.2 ± 0.4 ^a^	10.9 ± 0.8 ^a^
Kaempferol	19.9 ± 1.0 ^b^	18.4 ± 0.9 ^b^	98.1 ± 3.2 ^a^	23.2 ± 0.8 ^c^	31.2 ± 1.7 ^b^	78.6 ± 2.8 ^a^
Total flavonols	34.5	52.4	128.5	41.9	62.5	112.6
**Phenolic acid**						
*p*-coumaric	22.2± 1.5 ^c^	31.4 ± 2.0 ^b^	47.5 ± 2.9 ^a^	17.3 ± 1.0 ^b^	18.5 ± 1.4 ^b^	34.9 ± 2.7 ^a^
*p*-hydroxy-benzoic	33.6 ± 2.4 ^c^	52.1 ± 3.5 ^b^	65.4 ± 4.1 ^a^	21.4 ± 1.8 ^c^	38.9 ± 2.5 ^b^	47.9 ± 3.4 ^a^
Chlorogenic	11.5 ± 0.4 ^b^	12.7 ± 0.8 ^b^	18.2 ± 1.4 ^a^	14.2 ± 0.6 ^b^	16.9 ± 0.8 ^a^	12.2 ± 0.7 ^c^
Ferulic	8.4 ± 0.5 ^c^	12.5 ± 1.1 ^b^	17.3 ± 1.3 ^a^	7.6 ± 0.8 ^c^	12.5 ± 0.9 ^b^	24.1 ± 1.5 ^a^
Gallic	8.6 ± 0.5 ^c^	15.3 ± 1.4 ^b^	22.8 ±1.9 ^a^	6.8 ± 0.5 ^c^	10.2 ± 0.8 ^b^	24.6 ± 1.9 ^a^
Vanillic	ND	3.8 ± 0.1 ^b^	16.1 ± 1.0 ^a^	ND	2.8 ± 0.4 ^b^	11.8 ± 0.9 ^a^
∑ HBA	42.12	71.23	104.29	35.79	51.96	84.39
∑ HCA	42.07	56.65	82.93	38.98	47.88	71.11
∑ PHA	84.19	127.88	187.22	74.77	99.84	155.50

Values (mean ± SD) are averages of three samples of each fruit, analyzed individually in triplicate (*p* < 0.05); ND = not detected; Different letters in superscript represent significant differences of ripening stages; ∑ HBA = sum of benzoic acid derivatives; ∑ HCA = sum of cinnamic acid derivatives; ∑ PHA = sum of phenolic acids.

**Table 3:** Flavonols and phenolic acids composition (mg/100 g DW) of mulberry fruit at different maturity stages.

	*M. laevigata*			*M. macroura*		
**Flavonols**	Un-ripened	Semi-ripened	Fully-ripened	Un-ripened	Semi-ripened	Fully-ripened
Myricetin	11.5 ± 0.8 ^b^	22.3 ± 0.5 ^a^	20.0 ± 1.7 ^a^	11.5 ± 0.9 ^c^	19.2 ± 1.3 ^b^	22.5 ± 1.3 ^a^
Quercetin	7.0 ± 0.7 ^c^	88.8 ± 4.8 ^b^	145.7 ± 8.4 ^a^	7.0 ± 0.2 ^c^	13.9 ± 0.8 ^b^	21.7 ± 0.6 ^a^
Kaempferol	9.8 ± 0.6 ^c^	32.2 ± 2.5 ^b^	56.1 ± 3.9 ^a^	9.8 ± 0.6 ^a^	9.8 ± 0.3 ^a^	8.1 ± 0.4 ^b^
Total flavonols	28.3	143.3	221.8	28.3	42.9	52.3
**Phenolic acid**						
*p*-coumaric	ND	15.9 ± 1.1 ^b^	27.3 ± 2.5 ^a^	5.1 ± 0.7 ^b^	3.2 ± 0.4 ^c^	13.2 ± 1.3 ^a^
*p*-hydroxy-benzoic	1.1 ± 0.1 ^c^	4.1 ± 0.2 ^b^	7.3 ± 0.7 ^a^	5.1 ± 0.5 ^c^	17.5± 1.3 ^b^	24.1 ± 1.8 ^a^
Chlorogenic	3.4 ± 0.4 ^c^	7.1 ± 0.8 ^b^	12.9 ± 1.1 ^a^	4.2 ± 0.2 ^c^	13.2 ± 0.7 ^b^	23.2 ± 1.7 ^a^
Ferulic	12.4 ± 0.9 ^b^	ND	17.2 ± 0.9 ^a^	6.3 ± 0.3 ^b^	7.7 ± 0.5 ^b^	13.4 ± 1.1 ^a^
Gallic	5.2 ± 0.6 ^c^	8.8 ± 0.9 ^b^	14.2 ± 1.4 ^a^	4.2 ± 0.5 ^b^	3.5 ± 0.9 ^b^	9.8 ± 0.7 ^a^
Vanillic	8.5 ± 0.2 ^c^	13.3 ± 0.8 ^b^	21.1 ± 0.9 ^a^	3.2 ± 0.5 ^b^	1.9 ± 0.6 ^c^	16.1 ± 1.8 ^a^
∑ HBA	14.8	22.0	42.6	12.5	22.9	50.0
∑ HCA	15.8	26.2	57.4	15.6	24.1	49.8
∑ PHA	30.6	48.2	100.0	28.1	47.0	99.8
	***M. nigra***			***M. alba***		
**Flavonols**	Un-ripened	Semi-ripened	Fully-ripened	Un-ripened	Semi-ripened	Fully-ripened
Myricetin	52.6 ± 3.1 ^c^	63.3 ± 4.7 ^a^	56.1 ± 4.8 ^b^	45.0± 2.2 ^c^	78.1 ± 3.7 ^b^	88.4 ± 4.8 ^a^
Quercetin	8.1 ± 0.6 ^c^	43.5 ± 2.4 ^a^	11.7 ± 0.8 ^b^	1.3 ± 0.1 ^a^	1.3 ± 0.1 ^a^	0.7 ± 0.1 ^b^
Kaempferol	8.6 ± 0.7 ^c^	56.6 ± 3.5 ^a^	31.7 ± 1.9 ^b^	15.0± 0.1 ^a^	6.3 ± 0.1 ^b^	5.2 ± 0.2 ^b^
Total flavonols	69.3	163.4	99.5	61.3	85.7	94.3
**Phenolic acid**						
*p*-coumaric	4.2 ± 0.5 ^c^	7.6 ± 0.6^b^	21.2 ± 1.1 ^a^	1.1 ± 0.1 ^b^	2.0 ± 0.2 ^b^	4.0 ± 0.3 ^a^
*p*-hydroxy-benzoic	ND	ND	5.3 ± 0.5 ^a^	3.2 ± 0.5 ^b^	12.1 ± 1.2 ^a^	13.3 ± 1.2 ^a^
Chlorogenic	2.6 ± 0.5 ^b^	3.4 ± 0.2 ^b^	6.8 ± 0.1 ^a^	5.3 ± 0.4 ^c^	9.0 ± 0.7 ^b^	17.3 ± 1.8 ^a^
Ferulic	ND	2.4 ± 0.4 ^b^	7.5 ± 0.8 ^a^	ND	ND	ND
Gallic	2.5 ± 0.4 ^b^	3.9 ± 0.4 ^b^	8.3 ± 0.9 ^a^	3.6 ± 0.5 ^c^	6.2 ± 0.8 ^b^	8.1 ± 0.5 ^a^
Vanillic	6.1 ± 0.7 ^c^	10.2 ± 0.9 ^b^	18.3 ± 1.5 ^a^	1.7± 0.0 ^b^	2.2 ± 0.1 ^b^	5.7 ± 0.5 ^a^
∑ HBA	8.6	14.1	31.9	8.5	20.5	27.1
∑ HCA	6.8	13.4	35.5	6.4	11.0	21.3
∑ PHA	15.4	27.5	67.4	14.9	31.5	48.4

Values (mean ± SD) are averages of three samples of each fruit, analyzed individually in triplicate (*p* < 0.05); ND = not detected; Different letters in superscript represent significant differences in ripening stages; ∑ HBA = sum of benzoic acid derivatives; ∑ HCA = sum of cinnamic acid derivatives; ∑ PHA = sum of phenolic acids.

**Table 4:** Color/texture of strawberry and mulberry fruits at different maturity stages.

Fruits	Cultivar/Species	Un-ripened	Semi-ripened	Fully-ripened
Strawberry	Korona	Green/hard	Reddish green/semi-soft	Red/soft
	Tufts	Green/hard	Reddish green/semi-soft	Red/soft
Long mulberry	*M. laevigata*	Light Green/hard	Red/semi-soft	Black/soft
	*M. macroura*	Light Green/hard	Light yellow/semi-soft	Off-white/soft
Small mulberry	*M. nigra*	Light Green/hard	Red/semi-soft	Black/soft
	*M. alba*	Light Green/hard	Light yellow/semi-soft	Off-white/soft

## References

[1] Iriti M., Faoro F. (2006). Grape phytochemicals: A bouquet of old and new nutraceuticals for human health. Med. Hypotheses.

[2] Zhang Y., Seeram N.P., Lee R., Feng L., Heber D. (2008). Isolation and identification of strawberry phenolics with antioxidant and human cancer cell antiproliferative properties. J. Agric. Food Chem.

[3] Cordenunsi B.R., Genovese M.I., Do-Nascimento J.R.O., Aymoto-Hassimotto N.M., Santos R.J.D., Lajolo F.M. (2005). Effects of temperature on the chemical composition and antioxidant activity of three strawberry cultivars. Food Chem.

[4] Reganold J.P., Andrews P.K., Reeve J.R., Carpenter-Boggs L., Schadt C.W. (2010). Fruit and Soil Quality of Organic and Conventional Strawberry Agroecosystems. PLoS One.

[5] Harborne J.B., Baxter H., Moss G.P. (1999). Phytochemical dictionary. Handbook of Bioactive Compounds from Plants.

[6] Scalbert A., Williamson G. (2000). Dietary intake and bioavailability of polyphenols. J. Nutr.

[7] Lodovici M., Guglielmi F., Meoni M., Dolara P. (2001). Effect of natural phenolic acids on DNA oxidation *in vitro*. Food Chem. Toxicol.

[8] Robbins R. (2003). Phenolic acid in foods: An overview of analytical methodology. J. Agric. Food Chem.

[9] Wang S.Y., Zheng W. (2001). Effect of plant growth temperature on antioxidant capacity in strawberry. J. Agric. Food Chem.

[10] Ayala-Zavala J.F., Wang S.Y., Wang C.Y., Gonzalez-Aguilar G.A. (2004). Effect of storage temperatures on antioxidant capacity and aroma compounds in strawberry fruit. LWT Food Sci. Technol.

[11] Serrano M., Guillen F., Martinez-Romero D., Castillo S., Valero D. (2005). Chemical constituents and antioxidant activity of sweet cherry at different ripening stages. J. Agric. Food Chem.

[12] Patel P.R., Rao T.V.R. (2009). Physiological changes in relation to growth and ripening of khirni [*Manilkara hexandra* (Roxb.) Dubard] fruit. Fruits.

[13] Pineli L.L.O., Moretti C.L., Santos M.S., Campos A.B., Brasileiro A.V., Cordova A.C, Chiarello M.D. (2011). Antioxidants and other chemical and physical characteristics of two strawberry cultivars at different ripeness stages. J. Food Compos. Anal..

[14] Yang J., Gadi R., Thomson T. (2011). Antioxidant capacity, total phenols, and ascorbic acid content of noni (*Morinda citrifolia*) fruits and leaves at various stages of maturity. Micronesica.

[15] Shahidi F., Naczk M (2004). Phenolic compounds in fruits and vegetables. Phenolics in Food and Nutraceutical.

[16] Dinelli G., Bonetti A., Minelli M., Marotti I., Catizone P., Mazzanti A. (2006). Content of flavonols in Italian bean (*Phaseolus vulgaris* L.) ecotypes. Food Chem.

[17] Justesen U., Knethsen P. (2001). Composition of flavonoids in fresh herbs and calculation of flavonoids intake by use of herbs in traditional danish dishes. Food Chem.

[18] Mabberley D.J. (1987). The Plant-Book. A Portable Dictionary of the Higher Plants.

[19] Scott R.R., Lei Z., Tong W (2010). Assessments of Commodity and Trade Issues Made by USDA Staff and not Necessarily Statements of Official U.S. Government Policy; GAIN Report Number: 10043.

[20] Azodanlou R., Darbellay C., Luisier J.L., Villettaz J.C., Amado R. (2003). Quality assessment of strawberries (*Fragaria* species). J. Agric. Food Chem.

[21] Mitcham E.J., Gross K.C., Wang C.Y., Saltveit M.E. (2004). Strawberry. The Commercial Storage of Fruits, Vegetables, and Florist and Nursery Crops.

[22] Spayd S.E., Morris J.R. (1981). Physical and chemical characteristics of puree from once-over harvested strawberries. J. Amer. Soc. Hort. Sci.

[23] Terry L.A., Joyce D.C., Adikaram N.K.B., Khambay B.P.S. (2004). Preformed antifungal compounds in strawberry fruit and flower tissues. Postharvest Biol. Technol.

[24] Robards K., Antolovich M. (1997). Analytical chemistry of fruit bioflavonoids. A review. Analyst.

[25] Aherne S.A., OBbrien N.M. (2002). Dietary flavonols: chemistry, food content, and metabolism. Nutrition.

[26] Zadernowski R., Naczk M., Nesterowicz J. (2005). Phenolic acid profiles in small berries. J. Agric. Food Chem.

[27] Arabshahi-Delouee S., Urooj A. (2007). Antioxidant properties of various solvent extracts of mulberry (*Morus indica* L.) leaves. Food Chem.

[28] Ercisli S., Orhan E. (2007). Chemical composition of white *(Morus alba*), red *(Morus rubra*) and black (*Morus nigra*) mulberry fruits. Food Chem.

[29] Imran M., Talpur F.N., Jan M.S., Khan A., Khan I. (2007). Analysis of nutritional components of some wild edible plants. J. Chem. Soc. Pak.

[30] Sass-Kiss A. (2005). Differences in anthocyanin and carotenoids content of fruits and vegetables. Food Res. Int.

[31] Cieslik E., Greda A., Adamus W. (2006). Contents of polyphenols in fruit and vegetables. Food Chem..

[32] Lin J.Y., Tang C.Y. (2007). Determination of total phenolic and flavonoid contents in selected fruits and vegetables, as well as their stimulatory effects on mouse splenocyte proliferation. Food Chem.

[33] Kim D.O., Heo H.J., Kim Y.J., Yang H.S., Lee C.Y. (2005). Sweet and sour cherry phenolics and their protective effects on neuronal cells. J. Agric. Food Chem.

[34] Jakobek L., Seruga M., Medvidović-Kosanović M., Novak I. (2007). Anthocyanin content and antioxidant activity of various red fruit juices. Dtsch. Lebensm. -Rundsch.

[35] Jakobek L., Seruga M., Novak I., Medvidovic-Kosanovic M. (2007). Flavonols, phenolic acids and antioxidant activity of some red fruits. Dtsch. Lebensm. -Rundsch.

[36] Matilla P., Hellstrom J., Törrönen R. (2006). Phenolic acids in berries, fruits and beverages. J. Agric. Food Chem.

[37] Franke A.A., Custer L.J., Arakaki C., Murphy S.P. (2004). Vitamin C and flavonoid levels of fruits and vegetables consumed in Hawaii. J. Food Compos. Anal..

[38] Olsson M.E., Gustavsson K., Andersson S., Nilsson A., Duan R. (2004). Inhibition of cancer cell proliferation *in vitro* by fruit and berry extracts and correlations with antioxidant levels. J. Agric. Food Chem.

[39] Sultana B., Anwar F. (2008). Flavonols (kaempferol, quercetin, myricetin) contents of selected fruits, vegetables and medicinal plants. Food Chem.

[40] Bohm V., Kuhnert S., Rohm H., Scholze G. (2006). Improving the nutritional quality of microwave-vacuum dried strawberries: A preliminary study. Food Sci. Technol. Int.

[41] Piljac-Zegarac J., Samec D. (2011). Antioxidant stability of small fruits in postharvest storage at room and refrigerator temperatures. Food Res. Int.

[42] Bae S.H., Suh H.J. (2007). Antioxidant activities of five different mulberry cultivars in Korea. LWT Food Sci. Technol.

[43] Imran M., Khan H., Shah M., Khan R., Khan F. (2010). Chemical composition and antioxidant activity of certain *Morus* species. J. Zhejiang Univ. Sci. B.

[44] Gungor N., Sengul M. (2008). Antioxidant activity, total phenolic content and selected physicochemical properties of white mulberry (M*orus alba*. L.) fruits. Int. J. Food Prop.

[45] Scalzo J., Politi A., Pellegrini N., Mezzetti B., Battino M. (2005). Plant genotype affects total antioxidant capacity and phenolic contents in fruit. Nutrition.

[46] Hakkinen S.H., Torronen A.R. (2000). Content of flavonols and selected phenolic acids in strawberries and Vaccinium species: Influence of cultivar, cultivation site and technique. Food Res. Int.

[47] Aminah A., Anna P.K. (2011). Influence of ripening stages on physicochemical characteristics and antioxidant properties of bitter gourd (*Momordica charantia*). Int. Food Res. J.

[48] Ferreira I.C.F.R., Baptista P., Vilas-Boas M., Barros L. (2007). Free radical scavenging capacity and reducing power of wild edible mushrooms from northeast Portugal. Food Chem.

[49] Lugasi A., Hovari J. (2003). Antioxidant properties of commercial alcoholic and nonalcoholic beverages. Nahrung.

[50] Kevers C., Falkowski M., Tabart J., Defraigne J., Dommes J., Pincemail J. (2007). Evolution of antioxidant capacity during storage of selected fruits and vegetables. J. Agric. Food Chem.

[51] Hakkinen S., Heinonen M., Karenlampi S., Mykkanen H., Ruuskanen J., Torronnen R. (1999). Screening of selected flavonoids and phenolic acids in 19 berries. Food Res. Int.

[52] Kahkonen M.P., Hopia A.I., Vuorela H.J., Rauha J.P., Pihlaja K., Kujala T.S., Heinonen M. (1999). Antioxidant activity of plant extracts containing phenolic compounds. J. Agric. Food Chem.

[53] Maatta-Riihinen M., Kamal-Eldin A., Torronen A.R. (2004). Identification and quantification of phenolic compounds in berries of Fragaria and *Rubus* species (Family Rosaceae). J. Agric. Food Chem.

[54] Stohr H., Herrmann K. (1975b). The phenolics of fruits, the phenolics of strawberries and their changes during development and ripeness of the fruits. Z Lebensm-Unters Forsch.

[55] Kosar M., Kafkas E., Paydas S., Baser K.H.C. (2004). Phenolic composition of strawberry genotypes at different maturation stages. J. Agric. Food Chem.

[56] Ndri D., Calani L., Mazzeo T., Scazzina F., Rinaldi M., Rio D.D., Pellegrini N., Brighenti F. (2010). Effects of different maturity stages on antioxidant content of Ivorian Gnagnan (*Solanum indicum* L.) berries. Molecules.

[57] Vasco C., Riihinen K., Ruales J., Kamal-Eldin A. (2009). Chemical composition and phenoliccompound profile of mortiño (*Vaccinium* floribundum Kunth). J. Agric. Food Chem.

[58] Hernanz D., Recamales A.F., Melendez-Martinez A.J., Gonzalez-Miret M.L., Heredia F.J. (2007). Assessment of the differences in the phenolic composition of five strawberry cultivars (*Fragaria* ×*ananassa* Duch.) grown in two different soilless systems. J. Agric. Food Chem.

[59] Häkkinen S.H., Kärenlampi S.O., Mykkänen H.M., Törrönen A.R. (2000). Influence of domestic processing and storage on flavonol contents in berries. J. Agric. Food Chem.

[60] McDonald M., Hughes M., Burns J. (1998). Survey of the free and conjugated myricetin and quercetin content of red wines of different geographical origins. J. Agric. Food Chem.

[61] Lakenbrink C., Lapczynski S., Maiwald B., Engelhardt U.H. (2000). Flavonoids and other polyphenols in consumer brews of tea and other caffeinated beverages. J. Agric. Food Chem.

[62] Trichopoulou A., Vasilpoulou E., Hollman P. (2000). Nutritional composition and flavonoid content of edible wild greens and green pies: A potential rich source of antioxidant nutrients in the Mediterranean diet. Food Chem.

[63] Vuorinen H., Maata K., Torronen R. (2000). Content of the flavonols myricetin, quercetin, and kaempferol in Finnish berry wines. J. Agric. Food Chem.

[64] Soleas G.J., Diamandis E.P., Goldberg D.M. (1997). Resveratrol: a molecule whose time has come? And gone?. Clin. Biochem.

[65] Bilyk A., Sapers G.M. (1986). Varietal differences in the quercetin, kaempferol, and myricetin contents of highbush blueberry, cranberry, and thornless blackberry. J. Agric. Food Chem.

[66] Memon A.A., Najma M., Luthria D.L., Bhanger M.I., Pitafi A.A. (2010). Phenolic acids profiling and antioxidant potential of mulberry (*Morus laevigata* W., *Morus nigra* L., *Morus alba* L.) leaves and fruits grown in Pakistan. Pol. J. Food Nutr. Sci.

[67] Voca S., Dobricevic N., Dragovic-Uzelac V., Duralija B., Druzic J. (2008). Fruit quality of new early ripening strawberry cultivars in Croatia. Food Technol. Biotechnol.

[68] Jamil A., Anwar F., Ashraf M, Dris R. (2005). Plant Tolerance to Biotic and Abiotic Stresses through Modern Genetic Engineering. Crops, Growth, Quality and Biotechnology.

[69] Ali Q., Ashraf M. (2011). Exogenously applied glycine, betaine enhances seed and seed oil quality of maize (*Zea mays* L.) under water deficit conditions. Environ. Exp. Bot.

[70] Karjalainen R., Lehtinen A., Hietaniemi V., Pihlava J.M., Jokinen K., Keinänen M., Julkunen-Tiito R. (2002). Benzothiadiazole and glycine betaine treatments enhance phenolic compound production in strawberry. Acta Hortic.

[71] Chaovanalikit A., Wrolstad R.E. (2004). Total anthocyanins and total phenolics of fresh and processed cherries and their antioxidant properties. J. Food Sci.

[72] Dewanto V., Wu X., Adom K.K., Liu R.H. (2002). Thermal processing enhances the nutritional value of tomatoes by increasing total antioxidant activity. J. Agric. Food Chem.

